# Antifungal Activity of Copper Oxide Nanoparticles against Root Rot Disease in Cucumber

**DOI:** 10.3390/jof8090911

**Published:** 2022-08-28

**Authors:** Said M. Kamel, Samah F. Elgobashy, Reda I. Omara, Aly S. Derbalah, Mahmoud Abdelfatah, Abdelhamed El-Shaer, Abdulaziz A. Al-Askar, Ahmed Abdelkhalek, Kamel A. Abd-Elsalam, Tarek Essa, Muhammad Kamran, Mohsen Mohamed Elsharkawy

**Affiliations:** 1 Plant Pathology Research Institute, Agricultural Research Center, Giza 12619, Egypt; 2 Pesticides Chemistry and Toxicology Department, Faculty of Agriculture, Kafrelsheikh University, Kafr el-Sheikh 33516, Egypt; 3 Physics Department, Faculty of Science, Kafrelsheikh University, Kafr el-Sheikh 33516, Egypt; 4 Department of Botany and Microbiology, College of Science, King Saud University, P.O. Box 2455, Riyadh 11451, Saudi Arabia; 5 Plant Protection and Biomolecular Diagnosis Department, ALCRI, City of Scientific Research and Technological Applications, New Borg ElArab City 21934, Egypt; 6 School of Agriculture, Food and Wine, The University of Adelaide, Adelaide, SA 5005, Australia; 7 Agricultural Botany Department, Faculty of Agriculture, Kafrelsheikh University, Kafr el-Sheikh 33516, Egypt

**Keywords:** metal oxide nanoparticles, *Fusarium solani*, defense genes, control, resistance, enzymes, scanning electron microscope, anatomical structure

## Abstract

Metal oxide nanoparticles have recently garnered interest as potentially valuable substances for the management of plant diseases. Copper oxide nanoparticles (Cu_2_ONPs) were chemically fabricated to control root rot disease in cucumbers. A scanning electron microscope (SEM), X-ray diffraction (XRD) and photoluminescence (PL) were employed to characterize the produced nanoparticles. Moreover, the direct antifungal activity of Cu_2_ONPs against *Fusarium solani* under laboratory, greenhouse, and field conditions were also evaluated. In addition, the induction of host-plant resistance by Cu_2_ONPs was confirmed by the results of enzyme activities (catalase, peroxidase, and polyphenoloxidase) and gene expression (*PR-1* and *LOX-1).* Finally, the effect of Cu_2_ONPs on the growth and productivity characteristics of the treated cucumber plants was investigated. The average particle size from all the peaks was found to be around 25.54 and 25.83 nm for 0.30 and 0.35 Cu_2_O, respectively. Under laboratory conditions, the study found that Cu_2_ONPs had a greater inhibitory effect on the growth of *Fusarium solani* than the untreated control. Cu_2_ONP treatment considerably reduced the disease incidence of the root rot pathogen in cucumber plants in both greenhouse and field environments. Defense enzyme activity and defense genes (*PR1* and *LOX1*) transcription levels were higher in cucumber plants treated with Cu_2_ONPs and fungicide than in the untreated control. SEM analysis revealed irregularities, changes, twisting, and plasmolysis in the mycelia, as well as spore shrinking and collapsing in *F. solani* treated with Cu_2_ONPs, compared to the untreated control. The anatomical analysis revealed that cucumber plants treated with Cu_2_ONPs had thicker cell walls, root cortex, and mesophyll tissue (MT) than untreated plants. Cucumber growth and yield characteristics were greatly improved after treatment with Cu_2_ONPs and fungicide. To the best of our knowledge, employing Cu_2_ONPs to treat cucumber rot root disease is a novel strategy that has not yet been reported.

## 1. Introduction

In Egypt, cucumbers (*Cucumis sativus* L.) are among the most important cucurbitaceous crops and the leading export vegetable. Cucumbers are grown under protected cultivation conditions in plastic greenhouses in two main growing seasons, i.e., autumn and winter. Cucumbers are cultivated intensively under greenhouse conditions across a large area. Egypt ranks in 13^th^ place with regard to cucumber productivity across the world [1]. Cucumber is rich in vitamins A, B, and C and contains 96% water, 3% carbohydrates and 1% protein, minerals, such as manganese, copper, iron, calcium and potassium, and is low in calories [2]. Unfortunately, cucumber is susceptible to infection by several soil-borne fungi, causing damping-off and root rot diseases which, of course, affect the quality and productivity of the crop. *Fusarium solani* (Mart.) App. & Wr.; *Pythium ultimum* Trow; *Rhizoctonia solani* Khun and *Sclerotium rolfsii* Sacc. are considered the most important pathogens involved [3,4,5,6]. 

Some chemical fungicides are effective in controlling these diseases, but these chemicals are expensive and not eco-friendly. In addition, biological control is used as an easy-to-apply strategy and does not disrupt the ecological balance [7,8,9]. Nonetheless, there are some issues and challenges associated with the use of biological control, such as the high specificity between biological control agents and plant pathogens [10]. Furthermore, biological control materials must adapt to soil climate and crop conditions, not to mention the possibility of pathogen resistance, which reduces the effectiveness of biological control [11]. Based on all of the above, it has become very important to find non-conventional alternatives to control plant pathogens. 

The use of nanomaterials is one of the unconventional strategies for combating plant pathogens, as their unique and unusual physical, chemical, and biological properties have recently drawn the attention of members of the scientific community for their potential for a variety of purposes, including the control of plant pathogens. A straightforward, unambiguous solution to the issues with disease management is provided by nanotechnology [12,13,14]. The scientific and industrial fields are being revolutionized by these innovations. In the case of using nanotechnology in agriculture, the process of creating the final formulations that ensure the optimal distribution of agrochemicals, nutrients, pesticides, and even growth regulators, to increase the efficiency of use is particularly promising [15]. Various types of metallic and metal oxide nanoparticles with antimicrobial properties have been fabricated [16,17,18]. Metal nanoparticles, containing magnesium oxide [19], copper [20], silver [21,22], iron [23], zinc oxide [24], and nickel oxide [18,25], have shown antimicrobial properties. 

Cu NPs have recently been predicted to be a key component in the next generation of nanomaterials due to their low cost [26]. Furthermore, due to their excellent efficacy against a wide spectrum of microbes, copper oxide (CuO) and copper oxide (Cu_2_O) are among the most extensively used antimicrobial agents [26,27,28,29]. CuO is less expensive than silver, can be combined easily with polymers, and has chemical and physical properties that are generally stable. Researchers are particularly interested in high-ionic-scale metal oxides with antimicrobial activity, such as CuO, because they can be produced with unusually large surface areas and distinctive crystal shapes that add to their increased potency [19].

Furthermore, copper is a necessary plant micronutrient that aids in plant growth and disease resistance. Furthermore, copper is required for the creation of key plant defense proteins, such as plastocyanin, peroxidase, and copper multiple oxidases, in response to pathogen infections [30]. These nonspecific immune responses to infection can protect against a wide range of diseases. Elmer and colleagues [31,32,33] were among the first to demonstrate that the presence and function of CuO NPs can affect plant nutrition and disease defense. Cu_2_ONPs have also been used as a nano-fertilizer to promote disease resistance in a variety of plant/disease systems, such as asparagus/fusarium crown and root rot. The findings indicate that Cu_2_ONPs can operate as a very effective Cu delivery agent, promoting disease suppression [33]. As a result, the primary purpose of this study was to look into the physiological, pharmacological, and anatomical effects of Cu_2_ONPs in the control of cucumber root rot disease.

Induced resistance has been reported in viral, bacterial, and fungal diseases [18]. It offers an attractive alternative to genetic resistance, particularly for the control of diseases caused by soil-borne pathogens, which are treated with chemical pesticides. One of the new strategies for controlling plant pathogens in different crops is the use of systemic acquired resistance (SAR) on a large scale [34].

Thus, the current study aims to carry out the following objectives: (1) to fabricate Cu_2_ONPs of unique size and shape, verify their direct antifungal activity against *F. solani* in vitro, (2) to evaluate their ability to induce systemic resistance against root rot disease in cucumber plants in greenhouse and field conditions, (3) to determine the mechanism of resistance induction through the expression of regulatory and defense genes and the activity of defense enzymes and finally, (4) to look into the impact of produced nanoparticles on some of the growth and production features of cucumbers that have been treated.

## 2. Materials and Methods

### 2.1. Fabrication of Cu_2_ONPs 

Cu_2_ONPs were chemically prepared using a low-cost precipitation method, where 1 M copper sulfate (CuSO_4_. 5H_2_O) was dissolved in 100 mL deionized water (DI), followed by the addition of 5 M sodium hydroxide (NaOH), which was also dissolved in 100 mL DI. The reaction occurs at room temperature, after which different quantities of glucose solution (C_6_H_12_O_6_H_2_O) (0.30, 0.35 M) were heated to 50 °C and dropped into the mixture to reduce CuO to Cu_2_O nanoparticles. The precipitates were collected and centrifuged, then washed five times with DI and ethanol, respectively, before being dried for 24 h at 100 °C.

### 2.2. Characterization of Cu_2_ONPs 

SEM (JSM-651OLV) and X-ray diffraction (XRD, Shimadzu 6000) were used to investigate the crystal structure and morphological properties of the Cu_2_ONPs, respectively. The samples’ optical properties were investigated using a Kimmon He-Cd (325 nm) photoluminescence excitation laser, and the spectra were obtained using a HORIBA iHR320 spectrometer with a Synapse CCD camera. From XRD patterns, the particle size can be calculated employing Scherrer’s formula [35], which is as follows:

D_avg_ = K (λ/β cosθ)

where K is a shape factor and is usually taken to be 0.94, λ is the wavelength of the incident X-ray (Cu Kα1, 0.15406 nm), θ is the angle of Bragg, and β is the total width at half maxima (FWHM) in radians. 

### 2.3. Plant Materials 

Cucumber (*Cucumis sativus* L.) seeds of cv. Beta alpha were obtained from the Horticulture Research Institute, Agricultural Research Center (ARC), Dokki, Giza, Egypt.

### 2.4. Fungal Pathogen Identification 

*F. solani* was isolated from naturally infected cucumber plants that displayed damping-off and root rot symptoms. Cucumber samples were collected from various cultivated areas in Egypt’s Giza Governorate. The isolated fungus was purified with the hyphal tip technique and identified using morphological and microscopic features [36]. The identification of fungal isolates was confirmed morphologically at the Plant Pathology Research Institute (PPRI), Agricultural Research Center (ARC), Giza, Egypt. The identified fungus was kept in a potato dextrose agar (PDA) medium at 4 ± 1 °C [37]. 

### 2.5. Pathogenicity Test

An inoculum of the pathogen *F. solani* was prepared by growing the isolate in autoclaved bottles containing (100 g sorghum, 50 g sand, and 80 mL water) and incubated at 25 ± 2 °C. The sandy loam soil was autoclaved at 121 °C for 2 h. Plastic pots (30 cm in diameter) were sterilized using 5% formalin and left for 2 days to ensure complete formalin evaporation. Soil infestation was carried out by adding the previous inoculum to each pot at the rate of 3% of the soil weight. Five surface-sterilized cucumber seeds were sown in each pot seven days after soil infestation under controlled greenhouse conditions (25 ± 2 °C, 65 ± 2% humidity). Five replicates were used for each treatment, and pots with pathogen-free sandy loam soil were used for planting a control treatment. The percentage of damping-off at 15, 30, and 45 days after sowing was recorded.

### 2.6. Effects of Cu_2_ONP Concentrations on Fungal Growth 

The fungal isolate was cultured on a PDA medium for 7 days, then plugs (5 mm) were re-cultured again onto new PDA plates (9 mm), treated with two concentrations (50 and 100 µg/L) of 0.30 and 0.35 M Cu_2_O NPs, respectively. The fungicide Uniform 390 SE (azoxystrobin + mefenoxam), produced by Syngenta company, Basel, Switzerland, was used as a recommended fungicide against soil-borne disease at an application rate of 1.5 L/hectare). Five plates served as duplicates for each treatment. Plugs (5 mm) of *F*. *solani* grown on PDA were used as a control.

### 2.7. In Vivo (Greenhouse) Experiment

Cucumber seeds were planted at a rate of 5 seeds per pot. Five pots were used for each treatment. Infested soil with the pathogenic agent inocula was prepared as mentioned above in the section of the pathogenicity test. All pots were irrigated three times throughout the seven days. In this experiment, 0.30 and 0.35 M Cu_2_ONPs were applied at a concentration level of 100 µg/L, and the fungicide was used at 1.5 L/hectare. All treatments were applied by immersing the seeds for 60 min in the solution before sowing. The experimental design was a randomized complete block. The greenhouse conditions were 24 °C and 60% humidity. The percentages of pre-and post-emergence damping-off and survived plants were calculated according to Shaban and El-Bramawy [38], while root rot assessment was recorded according to the scale 0-4 of Hwang and Chang [39], with minor modifications, which were as follows: 0 = healthy roots, 1 = 1–9%, 2 ≥ 9–39%, 3 ≥ 39–69% and 4 ≥ 69% and above for root discoloration. Root discoloration was recorded at the end of the experiment and calculated according to the following formula:

Disease incidence (DI%) = number of infected plants/total plants in the treatment × 100

Root rot index = (total of all ratings/(total number of plants × 4)) × 100

### 2.8. Laboratory Studies

#### 2.8.1. Enzyme Activity Assay

To evaluate the effect of the tested treatments on the activity of defense enzymes (catalase, peroxidase, and polyphenol oxidase) in the treated cucumber plants, 0.5 g of freshly treated cucumber leaves were homogenized at 0–4 °C in 3 mL of 50mM TRIS buffer (pH 7.8), containing 1mM EDTA-Na_2_ and 7.5% polyvinylpyrrolidone. The homogenates were centrifuged (12,000 rpm, 20 min, 4 °C), and the enzyme activity was estimated at 25 °C, using a typical UV-160A spectrophotometer. Catalase (CAT), peroxidase (POX), and polyphenol oxidase (PPO) activities were measured, as demonstrated by Aebi [40], Hammerschmidt et al. [41], and Malik and Singh [42], respectively. 

#### 2.8.2. RT-PCR Analysis 

One week after germination, a 100 mg sample of cucumber leaf tissue was taken from the treated and control plants. With a pre-chilled mortar and pestle, the samples were immediately crushed in liquid nitrogen. Total RNA was extracted as explained by Tek and Calis [43]. The isolated RNA was utilized for qRT-PCR amplification. All qRT-PCRs were performed with real-time PCR equipment using the SYBR green method. The specificity was tested by creating a melting curve by progressively increasing the temperature to 95 °C. The gene-specific primers (*PR-1* and *LOX-1*) were used in quantitative RT-PCR (Table 1). To normalize the transcript levels for each sample, the actin gene was used as a reference gene, and the final data were calculated using the formula 2^−ΔΔCT^ [44].

#### 2.8.3. Microscopic Observations of Fungal Morphology

A light microscope (Leica DM1000) examination was used to study the effect of Cu_2_ONPs on mycelia and spores of *F. solani*. To study the interaction between *F. solani,* the cause of damping-off and root rot of cucumber plants and copper oxide nanoparticles Cu_2_ONPs (0.35) and Cu_2_ONPs (0.30), small pieces of agar were cut at the parts embedded with copper oxide nanoparticles with *F. solani* growth and transferred for dehydrating and were subsequently sputter-coated with gold according to methods of Harley and Ferguson [45]. Examination and photographing were carried out using a scanning electron microscope (SEM), JEOL JSM 6510 Iv, at the Faculty of Agriculture, Mansoura University, to observe the copper oxide nanoparticles’ effects through parasitism action. 

#### 2.8.4. The Anatomical Structure of Cucumber Plants

The anatomical structure of the median internode of the main roots and the grafting zone of 40-day old cucumber plants infected with *F. solani* and treated with 0.30 and 0.35 M Cu_2_ONPs were investigated. The samples were sliced and fixed in a solution made up of 10 mL formalin, 5 mL glacial acetic acid, and 85 mL 70% ethyl alcohol. Following that, the samples were washed in 50% ethyl alcohol, dried in a standard butyl alcohol series, embedded in paraffin wax at 56 °C (melting point), and cut with a rotary microtome. Finally, crystal violet and erythrosine prepared in Canada balsam were used to stain the samples [46]. An optical microscope was used to examine the slides, and counts and measures (m) for various tissues were calculated. 

### 2.9. Field Experiment 

A field experiment was conducted to evaluate the efficiency of Cu_2_ONPs and the recommended fungicide against the damping-off disease of cucumber. The experiment was designed in a randomized complete block with four replicates. The field in which the experiment was conducted has a previous history of the disease. Whether it was 0.30 or 0.35 M, Cu_2_ONPs were used at a rate of 100 μg/L and the fungicide at 1.5 L/ha. Each replicate was 2 × 10 m^2^ in the area and had two rows of 1 m in width and 10 m in length. Then, the soil was irrigated for 7 days before sowing. Cucumber seeds (cv. Beta Alpha) were planted at a spacing of 50 cm at a rate of 3 seeds per hole (after soaking in the tested treatments for 1 h). The effectiveness of the treatments in lowering damping-off and disease incidence in pre-and post-emergent stages, as well as the percentages of healthy plants that survived, were recorded after 15, 30, and 45 days. The percentages of pre-and post-emergence damping-off and survived plants were estimated according to the previously described method.

### 2.10. Growth and Yield Parameters

Plant height, root length, wet weight and dry weight were evaluated in both treated and untreated cucumber plants to evaluate the influence of the tested treatments on some growth characteristics of cucumber plants. According to Torres-Netto et al. [47], the total chlorophyll content in fully expanded cucumber leaves was determined using a portable paper chlorophyll meter (Minolta SPAD-502, Osaka, Japan). Shoot length was measured from the base of the cucumber plants to the top in centimeters; root length was measured from the base of cucumber plants to the top root in centimeters. Fresh weight was measured in grams and the dry weight of plants (80 °C for 36 h) was measured in grams when the weight was stable. Yield parameters, such as the number of fruits/plant, fruit weight (g), fruit weight/plant (kg), and % rate of increase yield, were measured.

### 2.11. Statistical Analysis

All experiments were designed with a complete randomized block design. The WASP software (Web Agriculture Stat Package) was used for the analysis of variance (ANOVA) at *p* ≤ 0.05. Using the SPSS v.22 software, analysis of variance and Pearson correlation tests were run to determine the relationship between all the measured parameters in this study.

## 3. Results

### 3.1. Characterization of Cu_2_ONPs

**Figure 1:**
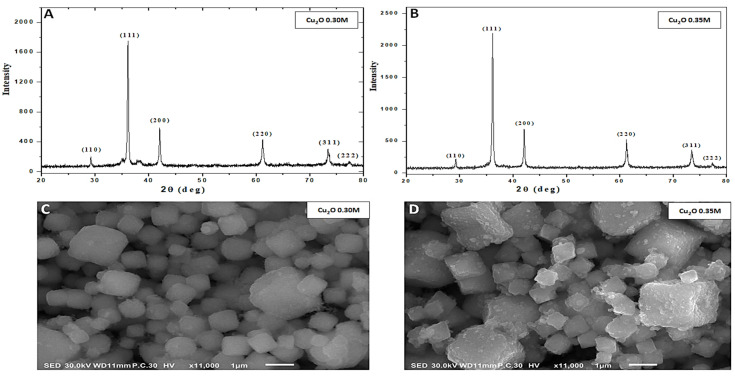
XRD patterns of the fabricated Cu_2_O nanoparticles (**A**,**B**). Top view of SEM image of surface morphologies of fabricated Cu_2_ONPs (**C**,**D**).

The crystal structure of the fabricated nanoparticles was examined by XRD, as presented in Figure 1A,B. The strongest peak of the fabricated Cu_2_O nanoparticles was formed at 2θ of around 36.43°, which is related to the (111) diffraction plane of the cubic phase of Cu_2_O [32]. There are also many peaks at 2θ of 29.58°, 42.32°, 61.39°, 73.54°and 77°, which correspond to the (110), (200), (220), (311) and (222) diffraction planes for the cubic phase structure of the Cu_2_ONPs, respectively [45,46]. All the observed diffraction peaks are associated with the standard polycrystalline cubic structure of Cu_2_O with the Pn3m group space (JPCD NO. 05-0667) [48,49]. Only a pure Cu_2_ONP phase is formed when no peaks for other phases appear. Moreover, the intensity (111) peak shows the high quality of crystallization. The calculated values for the particle size from the highest diffraction peak (111) were found to be around 30.96 and 31.91 nm for 0.30 and 0.35 Cu_2_ONPs, respectively. In addition, the average particle size from all the peaks was found to be around 25.54 and 25.83 nm for 0.30 and 0.35 Cu_2_O, respectively.

**Figure 2:**
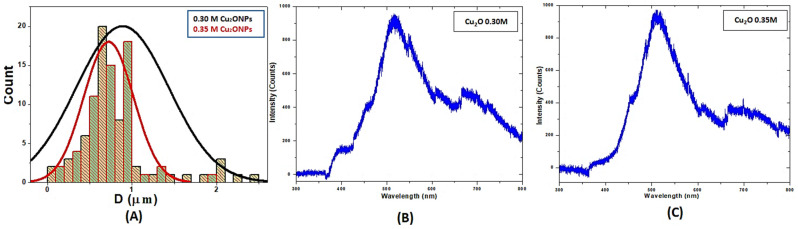
Average grain size distribution of 0.30 and 0.35 of Cu_2_O NPs (**A**) and room temperature PL spectra of fabricated Cu_2_ONPs (**B**,**C**).

Figure 1C,D display the top-view SEM images of the fabricated Cu_2_ONPs. It is noticeable that Cu_2_ONPs demonstrate the morphology of cubic structures [35]. The results confirm and agree with the XRD measurements. From SEM images, the distributions of grain size for the fabricated samples are calculated, as shown in Figure 2A. It is clear that the grain size for the 0.30 Cu_2_ONPs is smaller than the 0.35 Cu_2_ONPs, where its average values were around 0.7 and 0.9 µm, respectively. Therefore, the surface area of 0.30 is higher than 0.35. Such a change in the particle size, average grain size, and surface area for the Cu_2_O NPs is attributed mainly to the glucose amount during the reduction of CuO to Cu_2_ONPs, where glucose acts as a reduced agent and surfactant agent at the same time. In such a case, the increase in glucose prevents the agglomeration of the Cu_2_ONPs and increases the lateral and vertical growth, which produces smaller particle sizes and grain sizes for 0.30 Cu_2_ONPs than 0.35 Cu_2_ONPs [50,51].

Photoluminescence (PL) is a technique used to examine the recombination rate of produced electron–hole pairs and the quality of crystal for the fabricated materials [52,53]. Thus, PL spectra of the synthesized Cu_2_ONPs were described, as shown in Figure 2B,C. The PL spectrum has two emission peaks, which cover up the visible light region. The first one is broader and varies from around 360 to 610 nm and is centered at about 520 nm. The other has smaller intensity and less broadening and is centered at about 705 nm. Excitonic transition series of Wannier hydrogen-like electrons associated with deep-level defects are the responsible for the broad peak that can produce oxygen vacancies and/or copper interstitials [35]. This peak may be also due to the phonon-assisted excitations from the recombination process in nanoparticles [35]. The other peak resulted from the existence of other defects formed during the growth process, such as oxygen vacancies [35].

### 3.2. Antifungal Activity of Cu_2_ONPs against F. solani under Laboratory Conditions

**Figure 3:**
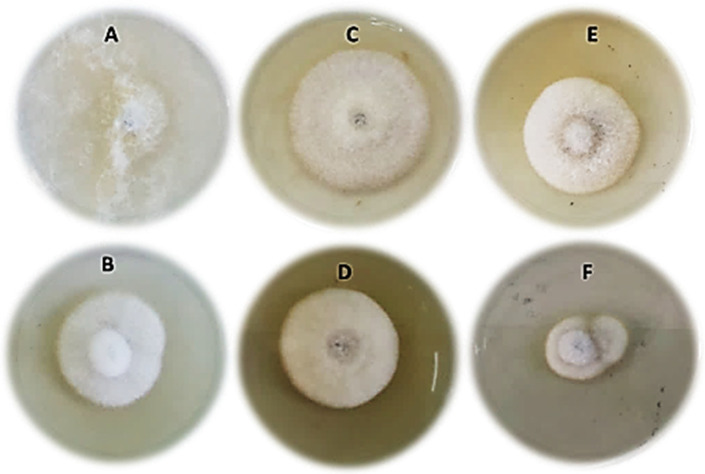
Effect of Cu_2_ONPs and chemical fungicide on the radial growth of *Fusarium solani* (**A**) = control, (**B**) = fungicide, (**C**) = Cu_2_ONPs (0.35 M) at a concentration of 50 µg/L, (**D**) = Cu_2_ONPs (0.35 M) at a concentration of 100 µg/L, (**E**) = Cu_2_ONPs (0.30 M) at a concentration of 50 µg/L and (**F**) = Cu_2_ONPs (0.30 M) at a concentration of 100 µg/L.

The effects of Cu_2_ONPs with its two molar concentrations, Cu_2_ONPs (0.35) and Cu_2_ONPs (0.30), on the mycelial growth of *F. solani* compared to the fungicide are presented in Table 2 and Figure 3. The results showed that the used Cu_2_ONPs and the fungicide significantly inhibited the growth of *F. solani* compared to the untreated control. The most effective treatment was the fungicide, followed by Cu_2_ONPs (0.30 M) and Cu_2_ONPs (0.35 M), respectively. In addition, there was a strong correlation between the inhibition percentage of the tested treatments and their concentrations.

### 3.3. Effect of Cu_2_ONPs on Disease Incidence under Greenhouse Conditions

The effect of Cu_2_ONPs compared to the recommended fungicide on the damping-off percentage and root rot disease incidence in the treated cucumber plants under greenhouse conditions is presented in Table 3. The results showed that 0.30 and 0.35 M of Cu_2_ONPs and the chemical fungicide significantly reduced the damping-off percentage and disease incidence in treated cucumber plants compared to the untreated control. The highest reduction in damping-off percentage and disease incidence was recorded for the recommended fungicide, followed by 0.30 and 0.35 M of Cu_2_ONPs, respectively. The reduction in the damping-off percentage and disease incidence in cucumber plants treated with 0.30 M of Cu_2_ONPs was significantly higher than that of 0.35 M of Cu_2_ONPs.

### 3.4. Effect of Cu_2_ONPs on the Activity of Defense Enzymes in Treated Cucumber Plants

The effect of Cu_2_ONPs and the recommended fungicide on the activity of defense enzymes in the treated cucumber plants is presented in Table 4. The data presented in Table 4 indicated that Cu_2_ONPs and the recommended fungicide significantly increased the activities of defense-related enzymes, i.e., catalase, peroxidase and polyphenol oxidase in treated cucumber plants compared to the untreated control. The highest activity for defense enzymes was recorded for 0.30 M Cu_2_ONPs, followed by 0.35 M Cu_2_ONPs and fungicide, respectively.

### 3.5. Relative Expression Assay

**Figure 4:**
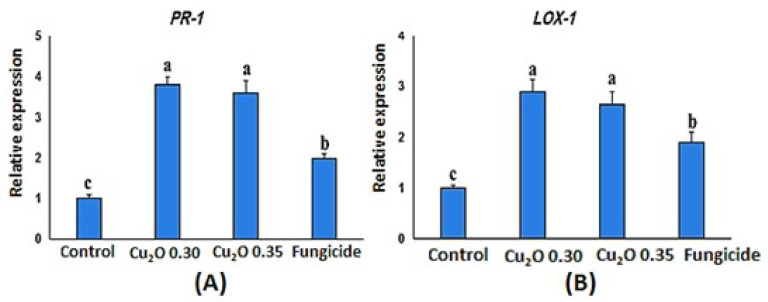
Expression of defense-related genes, such as *PR-1* (**A**) and *LOX-1* (**B**), in leaves of cucumber plants treated with Cu_2_ONPs before challenge inoculation with *F. solani*.

Defense gene transcriptions were substantially up-regulated among Cu_2_ONP treatments (Figure 4). Increased relative transcription levels of the *PR-1*, and *LOX-1* genes analyzed by qRT-PCR were observed in the treated cucumber plants at 7 days after treatment. It was revealed that in the Cu_2_ONP-treated plants, there were more up-regulated *PR-1* and *LOX-1* than in the control group, and that the Cu_2_ONP (0.30M)-treated plants had slightly higher *PR-1* and *LOX-1* transcription levels than the Cu_2_ONP (0.35 M) group. The relationship between the cucumber and *F. solani* was found to be mediated by some genes.

### 3.6. Laboratory Studies

#### 3.6.1. Microscopic Observations of Fungal Morphology

**Figure 5:**
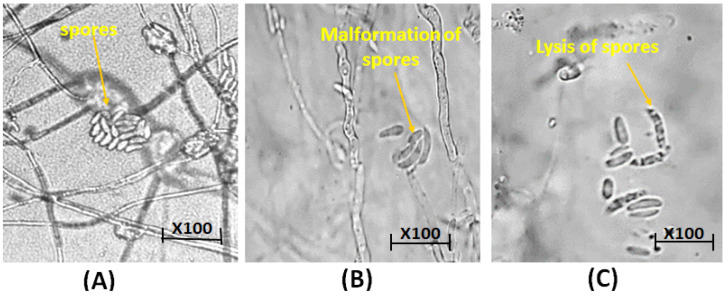
Light microscope observation of spores of *F. solani* showing (**A**): untreated control with normal spores and mycelium (yellow arrows). (**B**): Treated with Cu_2_ONPs (0.35M), showing collapsed spores (yellow arrows) and (**C**): treated with Cu_2_ONPs (0.30M), showing collapsed spores (yellow arrows).

Light microscope examinations of *F. solani* mycelia and spores treated with 0.30 and 0.35 M Cu_2_ONPs revealed shrinking, twisting, and collapse of treated plants (Figure 5B,C), compared to untreated *F. solani* (control) (Figure 4A).

**Figure 6:**
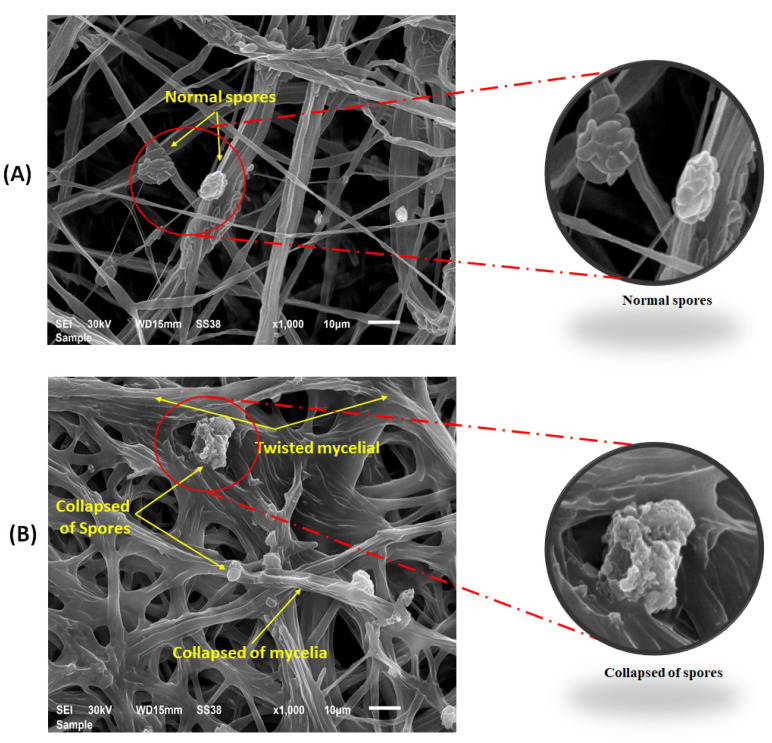
Scanning electron microscope observations of spores and mycelia of *F. solani* taken from growth medium on potato dextrose agar, with two sizes of nano copper showing. (**A**): Untreated control with normal spores and mycelium (yellow arrows). (**B**): Treated with Cu_2_ONPs, showing collapsed mycelia and spores (yellow arrows).

Scanning electron microscope examination of the fungal structures taken from cucumber roots treated with 0.30 and 0.35 M Cu_2_ONPs showed abnormalities and alterations in the mycelia of *F. solani* (Figure 6B), compared to the untreated control (Figure 6A). Moreover, twisting and plasmolysis of mycelial and spores and shrinking and collapsing were also observed in the roots of the treated cucumber plants (Figure 6B).

#### 3.6.2. The Anatomical Structure of Cucumber Plants

**Figure 7:**
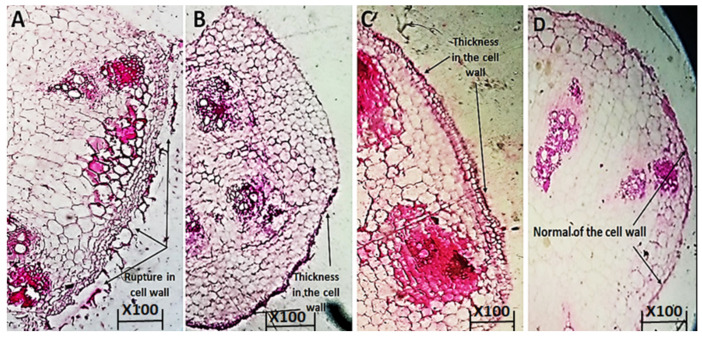
Effect of Cu_2_ONPs on the anatomical structure of cucumber root infection with *F. solani.* (**A**): infected control with *F. solani,* (**B**): Cu_2_ONPs (0.35 M), (**C**): Cu_2_ONPs (0.30 M) and (**D**): uninfected.

Our findings reveal that, in comparison to the untreated plants, cucumber plants treated with 0.30 and 0.35 M of Cu_2_ONPs increased the anatomical characters (Figure 7B,C). When plants were treated with 0.30 and 0.35 M Cu_2_ONPs, the cell wall, root cortex and mesophyll tissue (MT) thickness were all increased in comparison to the untreated plants, where the cell wall was ruptured (Figure 7A).

### 3.7. Effect of Cu_2_ONPs on Disease Incidence in Two Locations under Field Conditions

The effect of Cu_2_ONPs compared to the recommended fungicide on damping-off percentage and root rot disease incidence in cucumber plants in Menoufia and Giza Governorates is presented in Table 5. The results showed that 0.30 and 0.35 M of Cu_2_ONPs and the chemical fungicide significantly reduced the damping-off percentage and disease incidence in the treated cucumber plants compared to the untreated control in the two locations. The highest reduction in damping-off percentage and disease incidence was recorded for the recommended fungicide, followed by 0.30 and 0.35 M of Cu_2_ONPs in the two locations. The efficacy of Cu_2_ONPs and the recommended fungicide was higher in Giza than in Menoufia Governorate.

### 3.8. Effect of Cu_2_ONPs on Total Chlorophyll and Growth Parameter of Cucumber Plants under Field Conditions

The results in Table 6 indicated that the applied Cu_2_ONPs (0.30 and 0.35 M) and fungicide increased the growth parameters of treated cucumbers, such as total chlorophyll (SPAD), shoot length (cm), root length (cm), fresh and dry weight (g), under field conditions in the two locations compared to the untreated control. The highest growth parameters in the treated cucumbers were for the chemical fungicide, followed by 0.30 and 0.35 M of Cu_2_ONPs, respectively. Approximately, there are no significant differences in the measured growth parameters of cucumbers between Cu_2_ONPs 0.30 and 0.35 M in the two locations. The measured growth parameters of cucumber treated with Cu_2_ONPs and the fungicide were significantly higher in Giza Governorate than in the Menoufia Governorate.

### 3.9. Effect of Cu_2_ONPs on Yield Parameter of Cucumber Plants under Field Conditions

The results in Table 7 indicated that the applied Cu_2_ONPs (0.30 and 0.35 M) and fungicide increased the yield parameters of treated cucumbers, such as the number of fruits, fruits weight/plant, weight of fruits and rate of increased yield, under field conditions in the two locations compared to untreated control. The highest yield parameters in the treated cucumbers were for the chemical fungicide, followed by 0.30 and 0.35 M of Cu_2_ONPs, respectively. With regard to the measured yield parameters in the two locations, there are approximately no significant differences between Cu_2_ONPs (0.30) and the fungicide used. The measured yield parameters of the treated cucumbers were significantly higher in the cucumber plants treated with 0.30 M than with 0.35 M Cu_2_ONPs in the two locations.

### 3.10. Pearson Correlation

The relationship between the efficacy of the tested treatments and total chlorophyll, stem length (cm), root length (cm), fresh weight (g), dry weight (g), number of fruits, weight of fruits/plant, and rate of yield increase is indicated as shown in Table 8. The correlations between these variables were strongly positive and ranged between 0.904 and 0.998, which indicates the ability of these treatments under study to control this disease and increase growth and yield parameters.

## 4. Discussion

The pathogenic cucumber fungus *F. solani* is a common fungal genus that causes seed rot in cucumber seedlings, as well as pre-and post-emergency suppression of cucumber seedling production [54]. Cu_2_ONPs were examined in this work to control *F. solani* in cucumbers under laboratory, greenhouse and field conditions, and the results revealed a considerable reduction in the disease’s occurrence. In this investigation, copper oxide nanostructures were found to have promising antifungal action against *F. solani* under laboratory conditions. This is in agreement with [29], who found that copper oxide nanoparticles inhibited *F. solani* cultures significantly. Furthermore, the results agreed with those of Elmer and White [31], Elmer et al. [33], and Khatami et al. [29], who found that Cu_2_ONPs had a high potential for controlling soil borne fungi, such as *F. solani* and *F. oxysporum*. Consolo et al. [55] showed that both Ag and Cu_2_ONPs caused a significant reduction in the mycelia development of *A. alternata* and *P. oryzae* in a dose-dependent concentration. Moreover, copper compounds are still employed as fungicides to protect wood and prevent plant diseases [56,57]. From another point of view, copper oxide is a non-toxic, inorganic antimicrobial agent that inhibits the growth of a wide range of microbes [58,59,60,61,62]. The precise mechanism of copper oxide nanoparticles’ antimicrobial activity is still unknown. Researchers have proven in some published studies that the antibacterial activity may be related to the inactivation of the DNA enzyme, and as a result, impedes replication and growth inhibition [29]. Generally, some researchers discovered that NPs operate directly as antibacterial agents, while others discovered that their main role is to change the host’s nutritional status and activate defense mechanisms. Copper can be directly poisonous to microorganisms. Moreover, as fertilizer, Cu appears to contribute to host defense [33].

Accordingly, the obtained results show a significant reduction in the damping-off percentage and disease incidence in the cucumber plants treated with 0.30 and 0.35 M of Cu_2_ONPs under greenhouse and field conditions (Menoufia and Giza Governorates) compared to the untreated plants. As explained by some researchers, Cu_2_ONPs have high efficacy against several pathogens that cause damping-off and root rot, making them a viable alternative to fungicides in cucumber protection. It has a dual effect. The first is that it is directly toxic to diseases, and the second is that it can be used as a fertilizer, while also increasing the plant’s natural defenses against pathogens. Due to their long-standing usage as contact bactericides and fungicides, CuNPs are also an essential choice in plant disease management [27,31,63].

The induction of host-plant resistance by Cu_2_ONPs was confirmed by the results of enzyme activities (catalase, peroxidase, and polyphenoloxidase) and gene expression (*PR-1* and *LOX-1).* This was shown by the fact that Cu_2_ONPs caused an increment in the activity of catalase, peroxidase, and polyphenol oxidase, as well as in the gene expression of *PR-1,* and *LOX-1*. Elmer [33] provided an explanation for this by demonstrating how the presence of both Cu_2_ONPs and *F. oxysporum* f. sp. *niveum* strongly up-regulated the gene expression for *polyphenol oxidase* (*PPO*) and *PR1* in watermelon roots. The PPO enzyme assay results supported the gene expression findings. In order to effectively provide this micronutrient to fight disease, Cu_2_ONPs may be used. According to Ashraf et al. [64], treatment with varying concentrations of CuO-CFNPs resulted in an upward trend in photosynthetic pigments, phenolic content, and stress/antioxidant enzymatic components. In addition, *PR-1* is a gene that is often expressed if SAR activity is stimulated [65]. Similarly, transcriptions were increased in Cu_2_ONP treatments. The biosynthesis process of jasmonic acid, the phytohormone that controls ISR, begins with the synthesis of *LOX-1*, which is the first enzyme produced in this pathway [65].

The effect of Cu_2_ONPs in controlling the disease was also confirmed using a light microscope, a scanning electron microscope, and anatomical characteristics. The significant effect of Cu_2_ONPs was observed on mycelia and spores (abnormalities and alterations, twisting, plasmolysis, shrinking and collapsing) of *F. solani*. In addition, the cell wall, root cortex, and mesophyll tissue (MT) thickness were all increased with the treatment by Cu_2_ONPs, in comparison to the untreated plants, where the cell wall was ruptured. According to Ashraf et al. [64], comparative exposure to larger concentrations has severe negative consequences on the mycelial surface, resulting in split, distorted, and collapsed structures with tiny vesicles, similar to polyps. It was proposed that Cu-NPs released Cu ions into the growth media, which could diffuse past the cell wall and bond with the surface of fungal cells. Our findings unmistakably demonstrate that fungal mycelia become distorted following treatment with Cu_2_ONPs, which may be related to the interruption of chitin synthesis. Another interpretation revealed that the properties and chemical content of the nanoparticles, as well as their size and surface coating, when they interact with plants, cause several morphological and physiological changes [66].

These findings were in line with those of Elsharkawy et al. [67], who demonstrated that chitosan nanoparticles improved the thickness of the mesophyll tissue (MT), the thickness of the lower and upper epidermis (LE and UE), and the bundle length and width in the midrib in comparison to the control of treated wheat plants. Furthermore, Kim et al. [68] discovered that due to the disruption of the membrane integrity, *Candida albicans’* normal budding process was inhibited and the structure of the cell membrane was disrupted.

From another point of view, cucumber plants treated with Cu_2_ONPs and a fungicide grew better and demonstrated a better yield in the Governorates of Menoufia and Giza, according to this study. Elmer and White [31] found that foliar spraying of CuONPs substantially enhances root content and fresh weight in eggplants, compared to untreated eggplant. Elmer [33] demonstrated that CuONP-treated plants produced and yielded 39% more fruit than the untreated controls. Furthermore, the administration of Cu-NPs aided mung bean roots and shoot growth. According to Yasmine et al. [69], when wheat was treated with 25 ppm Cu-NPs, the spike length of wheat climbed slowly or remained unchanged, while the number of grains increased dramatically.

Copper compounds have been successful in the control of crop diseases caused by certain fungi and bacteria, as their cost is low and the risks of emergence of resistant strains of pathogens to these compounds are few or low, due to these compounds having more than one site of toxic action [70]. Particularly, copper nanoparticles cause a change in protein expression, which is the key to inhibiting microbial growth [71]. Although silver compounds have the same toxicity as copper compounds on plant pathogens, Cu_2_ONPs are more beneficial for use in the agricultural environment because they are less toxic than AgNPs. Cu_2_ONPs also have numerous modes of inhibitory action for microbial diseases [72], allowing them to be used as an alternative to chemical fungicides to control a variety of plant pathogens that infect plants. This would be extremely useful in decreasing the negative effects of fungicides, particularly on edible plants and fresh vegetables [73]. We also hope, in the future, to increase the study of this compound in terms of toxicity and the adoption of international companies and for it to reach the final product for its application at the field level among farmers.

## 5. Conclusions

Cu_2_ONPs inhibited the growth of *F. solani* in the laboratory, and they also reduced the disease incidence of the pathogen that causes cucumber root rot under greenhouse and field environments. They also improved cucumber growth and production characteristics. Defense enzyme activity and defense genes were expressed at higher levels in the cucumber plants treated with Cu_2_ONPs than in the untreated control. In comparison to the untreated plants, scanning electron microscopy and the anatomical investigation indicated anomalies, alterations, twisting in the mycelia, diminishing spores, and the collapse of *F. solani*, as well as increased cell wall, root cortex, and mesophyll tissue (MT) thickness of the cucumber plants.

## Figures and Tables

**Table 1:** Forward and reverse primers sequence for *PR-1, LOX-1* and *actin* genes.

Gene	Forward Primer (′5–′3)	Reverse Primer (′5–′3)
*PR-1*	TGCTCAACAATATGCGAACC	TCATCCACCCACAACTGAAC
*LOX-1*	CTCTTGGGTGGTGGTGTTTC	TGGTGGGATTGAAGTTAGCC
*Actin*	TGCTGGTCGTGACCTTACTG	GAATCTCTCAGCTCCGATGG

**Table 2:** Radial growth and inhibition percentage of the Cu_2_ONPs and fungicide against *F. solani* in vitro with regression equation and degree of correlation.

Treatment	Conc. (µg/L)	Radial Growth (mm)	Inhibition %	Regression Equation	R²
Cu_2_ONPs (0.35)	10	7.0 ^b^ ±0.54 *	22.22 ^f^ ± 0.56	Y = 1.7611x − 33.49	0.99
	25	5.8 ^c^ ± 0.66	35.56 ^e^ ± 0.76		
	50	4.6 ^d^ ±0.48	48.89 ^d^ ±0.73		
	100	2.3 ^d^ ± 0.52	74.44 ^b^± 0.86		
Cu_2_ONPs (0.30)	10	6.8 ^c^ ± 0.64	24.44 ^d^± 0.78	Y = 1.6779x − 36.244	0.981
	25	5.4 ^e^± 0.57	38.89 ^c^ ± 0.91		
	50	4.1 ^f^ ± 0.43	54.44 ^b^ ±0.93		
	100	1.9 ^f^ ± 0.39	78.89 ^a^± 1.12		
Fungicide (Uniform 390 SE)	10	6.3 ^d^ ± 0.67	30.0 ^d^ ± 0.71	Y = 1.627x − 43.269	0.983
	25	5.1 ^e^ ± 0.55	43.33 ^c^ ± 0.89		
	50	3.5 ^f^ ± 0.45	61.11 ^b^± 1.07		
	100	1.3 ^f^ ± 0.41	85.56 ^a^± 1.14		
Control	0	9.0 ^a^ ± 0.69	0.0 ^g^ ± 0.19		

Statistical comparisons were made among treatments within a single column. * The different letters represent significant differences using Fisher’s LSD test at *p* ≤ 0.05. Each mean value came from three replicates.

**Table 3:** Effect of Cu_2_ONPs compared to the chemical fungicide on the percentages of damping-off and root rot disease incidence of *F. solani* in cucumber plants under greenhouse conditions.

Treatment	Damping-Off %			Disease Incidence%	% Efficacy
	Pre-Emergence	Post-Emergence	Survival		
Cu_2_ONPs (0. 30)	13.3 ^c^ ± 0.68 *	6.0 ^c^ ± 0.26	80.7 ^a^ ±1.35	26.9 ^c^ ± 0.57	50.0
Cu_2_ONPs (0.35)	30.0 ^a^ ± 0.89	0.0 ^d^ ± 0.02	70.0 ^c^ ± 1.23	28.6 ^b^ ± 0.63	46.8
Fungicide (Uniform 390 SE)	13.3 ^c^ ± 0.63	13.3 ^b^ ± 0.46	73.4 ^b^ ± 1.41	20.0 ^d^ ± 0.48	62.8
Control	17.5 ^b^ ± 0.47	17.5 ^a^ ± 0.53	65.0 ^d^ ± 1.12	53.8 ^a^ ± 0.63	0.0

Statistical comparisons were made among treatments within a single column. * The different letters represent significant differences using Fisher’s LSD test at *p* ≤ 0.05. Each mean value came from three replicates.

**Table 4:** Effect of Cu_2_ONPs compared to the chemical fungicide on enzyme activities in treated cucumber plants.

Treatments	Enzyme Activity		
	CAT (Catalase) mM H_2_O_2_ g^−1^ FW Min^−1^	POX (Peroxidase) mM H_2_O_2_ g^−1^ FW Min^−1^	PPO (Polyphenol Oxidase) µ mol/min^−1^ g^−1^ (FW)
Cu_2_ONPs (0.30)	23.3 ^a^ ± 0.63 *	1.397 ^a^ ± 0.23	0.127 ^a^ ± 0.52
Cu_2_ONPs (0.35)	22.1 ^b^ ± 0.61	1.239 ^a^ ± 0.28	0.098 ^b^ ± 0.43
Fungicide (Uniform 390 SE)	19.7 ^c^ ± 0.46	0.741 ^b^ ± 0.25	0.054 ^c^ ± 0.35
Control	12.8 ^d^ ± 0.42	0.329 ^c^ ± 0.21	0.034 ^d^ ± 0.22

Statistical comparisons were made among treatments within a single column. * The different letters represent significant differences using Fisher’s LSD test at *p* ≤ 0.05. Each mean value came from three replicates.

**Table 5:** Effect of Cu_2_ONPs compared to the fungicide on the percentages of damping-off and root rot disease incidence of *F. solani* in cucumber plants under field conditions in Menoufia and Giza Governorates.

Treatments	Menoufia Governorate				
	Damping-Off %			Disease Incidence %	% Efficacy
	Pre-Emergence	Post-Emergence	Survival		
Cu_2_ONPs (0.35)	12.0 ^b^ ± 0.65	18.7 ^c^ ± 0.68	69.3 ^b^ ± 1.23	24.7 ^b^ ± 0.54	63.8
Cu_2_ONPs (0.30)	9.3 ^c^ ± 0.56	10.3 ^b^ ± 0.54	80.4 ^a^ ± 1.41	21.9 ^c^ ± 0.46	67.9
Fungicide (Uniform 390 SE)	7.3 ^c^ ± 0.51	11.3 ^b^ ± 0.57	81.4 ^a^ ± 1.46	18.0 ^d^ ± 0.44	73.6
Control	14.5a ± 0.72	29.2 ^a^ ± 0.81	56.3 ^c^ ± 1.27	68.3 ^a^ ± 1.75	0.0
	Giza Governorate				
Cu_2_ONPs (0.35)	9.7 ^b^ ± 0.58	12.7 ^c^ ± 0.59	77.6 ^c^ ± 1.34	21.3 ^b^ ± 0.58	70.17
Cu_2_ONPs (0.30)	7.4 ^c^ ± 0.51	11.3 ^b^ ± 0.56	81.6 ^b^ ± 1.42	20.4 ^b^ ± 0.66	71.43
Fungicide (Uniform 390 SE)	5.7 ^c^ ± 0.47	10.3 ^b^ ± 0.51	84.0 ^a^ ± 1.44	17.3 ^c^ ± 0.51	75.77
Control	13.5 ^a^ ± 0.62	31.7 ^a^ ± 0.74	54.8 ^d^ ± 1.04	71.4 ^a^ ± 1.42	0.0

Statistical comparisons were made among treatments within a single column. * The different letters represent significant differences using Fisher’s LSD test at *p* ≤ 0.05. Each mean value came from three replicates.

**Table 6:** Effect of Cu_2_ONPs compared to the chemical fungicide on total chlorophyll and growth parameters of cucumber plants under field conditions in Menoufia and Giza Governorates.

Treatment	Total Chlorophyll (SPAD)	Shoot Length (cm)	Root Length (cm)	Fresh Weight (g)	Dry Weight (g)
Menoufia Governorate					
Cu_2_ONPs (0.35)	32.1 ^b^ ± 0.74	176.4 ^a^ ± 1.21	30.8 ^b^ ± 0.85	45.2 ^a^ ± 0.67	4.8 ^b^ ± 0.23
Cu_2_ONPs (0.30)	33.7 ^b^ ± 0.77	177.2 ^a^ ± 1.24	31.1 ^b^ ± 0.82	47.7 ^b^ ± 0.86	5.2 ^b^ ± 0.23
Fungicide (Uniform 390 SE)	35.5 ^a^ ± 0.71	178.8 ^a^ ± 1.32	32.9 ^a^ ± 0.79	47.3 ^a^ ± 0.90	6.7 ^a^ ± 0.31
Control	23.4 ^c^ ± 0.59	130.3 ^b^ ± 1.21	23.2 ^c^ ± 0.98	29.3 ^c^ ± 0.47	3.3 ^c^ ± 0.22
Giza Governorate					
Cu_2_ONPs (0.35)	35.7 ^a^ ± 0.73	181.3 ^b^ ± 1.34	31.2 ^b^ ± 0.79	48.9 ^a^ ± 0.76	6.1 ^a^ ± 0.43
Cu_2_ONPs (0.30)	36.1 ^a^ ± 0.74	182.4 ^a^ ± 1.43	33.2 ^a^ ± 0.88	49.3 ^a^ ± 0.56	6.4 ^a^ ± 0.46
Fungicide (Uniform 390 SE)	36.5 ^a^ ± 0.68	179.3 ^b^ ± 1.44	33.9 ^a^ ± 0.81	50.7 ^a^ ± 0.67	6.3 ^a^ ± 0.42
Control	24.4 ^b^ ± 0.42	131.8 ^c^ ± 1.07	25.1 ^c^ ± 0.57	28.1 ^b^ ± 0.37	3.5 ^c^ ± 0.32

Statistical comparisons were made among treatments within a single column. * The different letters represent significant differences using Fisher’s LSD test at *p* ≤ 0.05. * Each mean value came from three replicates.

**Table 7:** Effect of Cu_2_ONPs compared to the chemical fungicide on yield parameters of cucumber plants under field conditions in Menoufia and Giza Governorates.

Treatments	No. of Fruits	Mean Weight of Fruits (g)	Fruits Weight/Plant (kg)	% Rate of Yield Increase **
Menoufia Governorate				
Cu_2_ONPs (0.35)	27.7 ^b^ ± 0.78 *	73.4 ^b^ ± 0.99	2.033 ^b^ ± 0.34	96.4 ^c^ ± 1.03
Cu_2_ONPs (0.30)	28.3 ^a^ ± 0.76	74.2 ^a^ ± 0.95	2.099 ^b^ ± 0.37	102.8 ^b^ ± 1.11
Fungicide (Uniform 390 SE)	29.4 ^a^ ± 0.79	74.8 ^a^ ± 0.97	2.199 ^a^ ± 0.29	112.5 ^a^ ±1.14
Control	14.3 ^c^ ± 0.49	72.4 ^c^ ± 0.93	1.035 ^c^ ± 0.19	0.00 ^d^ ± 0.78
Giza Governorate				
Cu_2_ONPs (0.35)	28.3 ^b^ ± 0.89	74.1 ^a^ ± 0.94	2.097 ^b^ ± 0.37	89.7 ^c^ ± 0.99
Cu_2_ONPs (0.30)	29.4 ^a^ ± 0.87	74.9 ^a^ ± 0.98	2.202 ^a^ ± 0.43	99.3 ^b^ ± 0.94
Fungicide	29.7 ^a^ ± 0.84	75.2 ^a^ ± 1.04	2.233 ^a^ ± 0.34	102.1^a^ ± 1.05
Control (Uniform 390 SE)	15.4 ^c^ ± 0.53	71.8 ^c^ ± 0.97	1.105 ^c^ ± 0.29	0.0 ^d^ ± 1.01

Statistical comparisons were made among treatments within a single column. * The different letters represent significant differences using Fisher’s LSD test at *p* ≤ 0.05. Each mean value came from three replicates. ** Rate of yield increase = (fruit weight/plant (treatment) − fruit weight/plant (control))/(fruit weight/plant (control)) × 100.

**Table 8:** Pearson correlations between efficacy and total chlorophyll and growth parameters and yield parameters.

	Efficacy	Total Chlorophyll	Shoot Length	Root Length	Fresh Weight	Dry Weight	No. of Fruits	Mean Weight of Fruits	Fruits Weight/Plant	Rate of Yield Increase
Efficacy	1	0.978 **	0.995 **	0.972 **	0.989 **	0.914 **	0.998 **	0.923 **	0.998 **	0.991 **
Total chlorophyll		1	0.976 **	0.978 **	0.987 **	0.965 **	0.978 **	0.921 **	0.980 **	0.952 **
Shoot length			1	0.961 **	0.988 **	0.904 **	0.995 **	0.896 **	0.993 **	0.983 **
Root length				1	0.963 **	0.943 **	0.979 **	0.941 **	0.983 **	0.960 **
Fresh weight					1	0.917 **	0.986 **	0.911 **	0.986 **	0.968 **
Dry weight						1	0.914 **	0.922 **	0.920 **	0.888 **
No. of fruits							1	0.916 **	1.000 **	0.992 **
Mean weight of fruits								1	0.927 **	0.904 **
Fruits weight/plant									1	0.991 **
Rate of yield increase										1

** = highly significant.

## Data Availability

Not applicable.
